# Assessment of antibiotic appropriateness at discharge: experience from a quaternary care hospital setting

**DOI:** 10.1093/jacamr/dlac065

**Published:** 2022-07-11

**Authors:** Joanna Saleh, Wasim S El Nekidy, Rania El Lababidi

**Affiliations:** Department of Pharmacy, Cleveland Clinic Abu Dhabi, P.O. Box 112412, Abu Dhabi, United Arab Emirates; Department of Pharmacy, Cleveland Clinic Abu Dhabi, P.O. Box 112412, Abu Dhabi, United Arab Emirates; Cleveland Clinic Lerner College of Medicine of Case Western Reserve University, Cleveland, OH, USA; Department of Pharmacy, Cleveland Clinic Abu Dhabi, P.O. Box 112412, Abu Dhabi, United Arab Emirates

## Abstract

**Background:**

There is a gap in antimicrobial stewardship in transitions of care.

**Objectives:**

To assess the appropriateness of antibiotics utilized and prescribing habits at hospital discharge.

**Methods:**

A retrospective, observational study was conducted at our quaternary care hospital between January 2021 and March 2021. During the study period, all patients discharged on antibiotics for pneumonia (PNA), skin and soft tissue infections (SSTI), urinary tract infections (UTI) and intra-abdominal infections (IAI) were included. The overall appropriateness of therapy was assessed based on the following combined criteria: agent, dose, frequency, duration of therapy, and ability to meet diagnostic criteria.

**Results:**

One hundred and forty-five subjects met the inclusion criteria. Of these, 44 (30.3%) were determined to have received overall appropriate antibiotic therapy. The most common infections were UTI, followed by IAI, PNA, and SSTI, respectively. Further, from the group deemed to have received overall inappropriate therapy, 26 of the 101 (25.7%) patients received an inappropriate antibiotic choice, 6 (5.9%) an inappropriate dose, and 84 (83.2%) an inappropriate duration of therapy.

**Conclusions:**

Inappropriate duration of therapy represented the most challenging problem with antibiotic regimens at discharge. Larger studies are needed to identify potential interventions that are effective, and can be implemented in all settings, including resource-limited ones.

## Introduction

The misuse and overuse of antibiotics has led to the emergence of antimicrobial-resistant organisms.^1^ The introduction of antimicrobial stewardship programmes (ASPs) has greatly contributed to promoting the appropriate use of antibiotics. However, an area where there is a gap in stewardship is upon the transition of care, specifically at hospital discharge.^2^ This transition is a critical point in the patient’s journey and could contribute to patient re-admissions, complications, and increased financial burden on the patient as well as the healthcare system.^3^ Given the paucity of literature on the appropriateness of antimicrobial use at transitions of care in our region, we sought to conduct this study to assess the overall appropriateness of antibiotic regimens at our hospital in pneumonia (PNA), skin and soft tissue infection (SSTI), urinary tract infection (UTI), and intra-abdominal infection (IAI) and at discharge. This will also help us compare our findings with international data.

## Patients and methods

The study was a retrospective, observational study conducted at our quaternary care hospital between January 2021 and March 2021. Data collection was commenced after the receipt of the Research Ethics Committee approval. A Diagnosis Related Group (DRG) data extraction method for the targeted indications was used, and all data were collected from our electronic medical record system. Demographics, baseline characteristics, infection parameters, definitive therapies, length of hospital stay, and outcomes were collected.

A convenience sample of patients who were discharged during the study period and met the inclusion criteria was used. We included adults (≥18 years) being discharged from our hospital on antibiotics for the indications of PNA, SSTI, UTI, and IAI. Pregnant women, age <18 years, HIV positive, and immunocompromised subjects were excluded. The primary endpoints of the study were to evaluate the overall appropriateness of the antimicrobial regimens used at discharge, as well as to identify the utilization and prescribing patterns of antibiotics for the targeted indications. Secondary endpoints included assessing re-admission within 30 days of discharge, development of resistance identified by repeated cultures (if done), evaluating the occurrence of *Clostridioides difficile* infection (*C. difficile* or CDI), and the recurrence or relapse of patients with same organisms (see Table 1 for definitions).

**Table 1.** Definitions of endpoints

Endpoint	Definition
CDI	Infection with *C. difficile* during hospitalization or during re-admission within 30 days of antibiotics administration.
MDR	Multidrug-resistant organisms, defined as pathogens not susceptible to at least one antibiotic in at least three classes to which the pathogen is generally expected to be susceptible.
Appropriate antibiotics	Prescribed regimen with the right antimicrobial agent, the right dose, for the right duration, and ability to meet diagnostic criteria. This is as per our institutional Antimicrobial Use Guidelines, IDSA guidelines, and as per the patient’s allergies and cultures and patient’s previous bacterial colonization history.
Re-admission	Re-admission to the hospital due to an infection within 30 days of previous infection in the same site.
Recurrence	Infection with the same bacteria within 14 days of discharge.
Relapse	Infection in the same site with a different bacterium within 30 days of discharge.
Resistance	Development of infection with bacteria resistant to 3 or more antibacterial drug classes within 30 days of antibiotic administration.

The overall appropriateness of antimicrobial therapy prescribed was judged based on the collective of appropriateness of each of the regimen’s components (selected agent, dose, frequency, duration, and ability to meet diagnostic criteria). Diagnostic criteria comprised any laboratory test values, imaging, microbiology, etc. necessary to confirm infection. Therapy appropriateness was assessed against established institutional published guidelines (see Table S1, available as Supplementary data at *JAC-AMR* Online) as well best-practice evidence, such as the Infectious Disease Society of America (IDSA) guidelines, and was adjudicated through a detailed review of two clinical pharmacy specialists trained in ASP and transitions of care.

### Statistical analysis

Descriptive statistics were used to summarize demographics and baseline characteristics. Normality of data distribution was assessed using the Shapiro–Wilks test and by visually checking each variable's distribution (histogram). Descriptive statistics, such as frequencies, medians and IQRs, were used to describe the demographic and outcomes of the study sample. Bivariate analyses to compare independent groups were conducted using Mann–Whitney U test for continuous variables and *χ*^2^ test or Fisher exact test were used to compare non-parametric and discrete data respectively. A two-tailed alpha of 0.05 or 95% CI was used as the criteria to determine statistical significance in this study. All statistical analyses were performed using SPSS statistical package version 28 for Windows (IBM, Armonk, New York).

## Results

We screened 741 patients who were discharged on oral antibiotics for inclusion, of them, 146 patients met the inclusion criteria. Of those, one patient was further excluded due to lack of information in the profile that would preclude appropriate assessment. Of the remaining 145 patients, 68 (46.9%) were female with a median age of 56 (34.5–73) years. Table 2 shows the sample demographics and baseline characteristics.

**Table 2.** Demographics and baseline characteristic of the study sample

Variable	All (*n *= 145)	Overall appropriate (*n *= 44)	Overall inappropriate (*n *= 101)	*P* value
Gender (female), *n* (%)	68 (46.9)	21 (47.7)	47 (46.5)	0.895
Age, years, median (IQR) (*n *= 142)	56 (34.5–73)	57.5 (39.25–73.5)	56 (32.25–73)	0.484
Diabetes, *n* (%)	81 (55.9)	22(50)	42 (41.6)	0.348
Hypertension, *n* (%)	25 (34.2)	25 (56.8)	48 (47.5)	0.303
Chronic kidney disease, *n* (%)	30 (20.7)	11 (25)	19 (18.8)	0.398
Liver disease, *n* (%)	2 (1.4)	1 (2.3)	1 (1)	0.516
Chronic artery disease, *n* (%)	34 (23.8)	12 (27.9)	22 (21.7)	0.447
History of MDRO, *n* (%)	5 (3.4)	1 (2.3)	4 (4)	0.520
Primary infection, *n* (%)				0.843
UTI	52 (35.9)	16 (36.4)	36 (35.6)	
PNA	30 (20.7)	9 (20.5)	21 (20.8)	
IAI	31 (21.4)	11 (25)	20 (19.8)	
SSTI	32 (22.1)	8 (18.2)	23 (23.8)	
Route at discharge (oral), *n* (%)	128 (97.7)	37 (94.9)	91 (98.9)	0.211

MDRO, multidrug-resistant organism; UTI, urinary tract infection; PNA, pneumonia; IAI, intrabdominal infections; SSTI, skin and soft tissue infection.

Table 3 summarizes the outcomes of the study endpoints. Forty-four (30.3%) patients received overall appropriate antimicrobial therapy versus 101 patients (69.7%) that received overall inappropriate regimens. Appropriate choice of antimicrobial was seen in 119 (82%) patients, the dose of antimicrobial was appropriate in 139 (96%) patients, and the duration of antimicrobial therapy was appropriate in 61 (42%) patients. Eighteen (12.4%) patients failed to meet diagnostic criteria. As far as the components of the inappropriate regimens, 26 of the 101 (25.7%) patients received the inappropriate antibiotic choice, 6 (5.9%) the inappropriate dose, and 84 (83.2%) the inappropriate duration of therapy. Duration of therapy was longer than recommended in treatment guidelines in all of the prescribed regimens.

**Table 3.** Treatment outcomes

Variable	All (*n *= 145)	Appropriate (*n *= 44)	Inappropriate (*n *= 101)	*P* value
Appropriate antimicrobial	119 (82.1)	44 (100)	75 (74.3)	<0.001
Appropriate dose	139 (95.9)	44 (100)	95 (94.1)	0.109
Appropriate duration	61 (42.1)	44 (100)	17 (16.8)	<0.001
Failure to meet diagnostic criteria	18 (12.4)	0 (0)	18 (17.8)	0.003
Readmission within 30 days	11 (7.6)	3 (6.8)	8 (7.9)	0.559
Recurrence within 14 days	5 (3.4)	1 (2.3)	4 (4)	0.520
Relapse within 30 days	6 (4.1)	2 (4.5)	4 (4)	0.591
Resistance development	1 (0.7)	1 (1.6)	0 (0)	0.299
CDI within 30 days	1 (0.7)	0 (0)	1 (1)	0.697

All values shown are *n* (%). CDI, *C. difficile* infection.

UTI represented the most infections with 16 (36.4%) subjects in the overall appropriate group versus 36 (35.6%) in the overall inappropriate group, followed by IAI, then PNA, and finally SSTI with no significant difference between groups (*P *= 0.843; Table 2).

In the definitive antimicrobial therapy at discharge, amoxicillin/clavulanic acid represented the most commonly prescribed antimicrobial. When comparing the two groups, amoxicillin/clavulanic acid was prescribed in 13 (26.6%) patients in the appropriate group versus 28 (27.7%) in the inappropriate group. This was followed by ciprofloxacin, the next most commonly prescribed antimicrobial. It was prescribed in 7 (16%) patients in the appropriate group versus 19 (19%) patients in the inappropriate group (*P *= 0.914). Majority of patients were initially started on IV antimicrobials, 39 (88%) in the appropriate group versus 87 (86%) in the inappropriate group (*P *= 0.129).

In the secondary outcomes (Table 3), 30 day readmissions occurred in 11 patients (7.6%), 8 of which were in the inappropriate group. Recurrence within 14 days occurred in 5 patients (3.4%), 4 of which were in the inappropriate group. Relapse within 30 days occurred in 6 patients (4.1%), 4 of whom were in the inappropriate group. Resistance development and CDI occurred in one patient each post-discharge, one in the appropriate group and the other in the inappropriate group. These were all not statistically significant, which could be explained by our small sample size.

## Discussion

Few studies have assessed antibiotic appropriateness at discharge, for instance, Vaughn *et al*.^4^ analysed the overuse of antibiotics at discharge for PNA or UTI in approximately 22 000 patients. They determined that fluoroquinolones were the most prescribed agents at discharge, with 49% of patients experiencing antimicrobial misuse.^4^ Similarly, Leja *et al*.^5^ retrospectively evaluated the impact of antimicrobial optimization in patients with high mortality risk at discharge, allowing pharmacist interventions to correct the regimens as they identified more than 2000 interventions. Further, Yogo *et al*.^6^ assessed the appropriateness of antibiotic regimens of 300 patients at discharge, finding that 53% of regimens were inappropriate, especially in PNA, UTI, and SSTI.

Our study was a retrospective analysis of patients discharged with the targeted indications mentioned above as we attempted to analyse the appropriateness of antimicrobial regimens prescribed at transitions of care. Based on our study findings, inappropriate duration of therapy represents the most common problem with antimicrobial regimens prescribed at discharge. One hundred and one subjects (70%) in our study population received an overall inappropriate therapy. This was due to either inappropriate antibiotic choice or, more commonly, inappropriate duration of therapy. This rate is in line with previous studies reporting on inappropriateness of therapy prescribed at transitions of care with recent studies reporting inappropriate therapy ranging from 26% to 47%.^7–9^ In our study, we found that treatment duration was excessively longer than what is currently recommended in treatment guidelines, which is similar to the findings of previous studies. Ensuring appropriate treatment duration is crucial and has been a focus of various recent studies investigating shortening durations of therapy and the positive impact it has on reducing development of antimicrobial resistance and/or *C. difficile* infections without compromising clinical outcomes.^10–13^

In our study, appropriate choice and appropriate dosing was found to be high in part because we have a robust ASP and published institutional guidelines and order-sets that drive appropriate choice/dosing of antimicrobials during the inpatient admission. Our institution is a 364 bed hospital, which is an extension of a US-based model of care in the Arabian Gulf region. Our ASP programme has been in place since March 2015 and is designated as a Center of Excellence by the IDSA. The programme is co-directed by a PGY2 trained Infectious Diseases pharmacist, who oversees both the day-to-day clinical activities and the ASP Subcommittee: a monthly forum that addresses antimicrobial formulary changes as well as updates to antimicrobial policies, protocols, and guidelines. There are several intermediate to advanced-level initiatives implemented through the programme, including prospective audit with intervention and feedback, prior authorization of broad-spectrum antimicrobials, facility-specific antimicrobial guidelines, real-time rapid diagnostics, real-time computerized surveillance system, and real-time dashboard for ASP metrics.^14,15^ In addition, members of the ASP are currently represented on the National Antimicrobial Resistance (AMR) taskforce. However, our programme has not yet focused on transitions of care and this is our first assessment of this under-recognized area within ASPs that is recently coming into the spotlight as a result of new research findings. Additionally, we do not have a formalized outpatient parenteral antimicrobial therapy (OPAT) service, however, the hospital works with various home healthcare agencies to assist with transition of care of patients on long-term IV antimicrobial therapy.

Some interventions that have been found to be effective in some medical centres include implementing a transitions of care team that specifically targets review of antimicrobials at transitions of care, and provides timely feedback to prescribers whenever issues are identified. Interventions addressing transitions of care would need to carefully weigh the institutional resources available and would need to be timely, so as to not impact throughput at discharge, which is another metric closely measured by most institutions.

The limitations of our study are the retrospective design, the relatively small sample size included (represents approximately 5% of total inpatient discharges during that time frame), and being from a single centre. Another limitation of our study is that it does not include the breakdown of durations of therapy (how many days in excess of recommendation were the inappropriately long durations), the spectrum of antibiotics when looking at inappropriate versus appropriate choice, and the breakdown of doses (too high versus too low) in each group.

### Conclusions

Our study highlights a global issue encountered with antimicrobial prescribing at discharge and corroborates previous research in this setting. Addressing appropriateness of antimicrobial prescribing through a systems approach is crucial, as it could potentially impact clinical outcomes including readmission rates, antimicrobial resistance development and development of antimicrobial adverse events including *C. difficile*. There is an urgent need for larger studies to be conducted in this setting to identify potential interventions that are effective and can be implemented in all settings, including resource-limited ones.

## Supplementary Material

dlac065_Supplementary_Data

## Supplementary data

Table S1 is available as Supplementary data at *JAC-AMR* Online.
