# Supplementary material for: Assessment of antibiotic appropriateness at discharge: experience from a quaternary care hospital setting
Source: JAC Antimicrob Resist. 2022 Jul 11;4(4):dlac065. doi: 10.1093/jacamr/dlac065 (PMC9271486; doi:10.1093/jacamr/dlac065)
Supplement: dlac065_Supplementary_Data [file dlac065_supplementary_data.docx]

**Supplementary data**

**Table S1.** Guidelines for the Treatment of Common Infectious Diseases^1^

| Clinical Setting | Empiric Therapy | Likely Pathogen | Directed Therapy | Usual Duration |  |
| --- | --- | --- | --- | --- | --- |
|  |  |  |  |  |  |
| Uncomplicated urinary tract infection, cystitis | Nitrofurantoin x 5 days  Fosfomycin x 2 doses | *E. coli*  Other Enterobacterales  *Staphylococcus saprophyticus* | Cefixime  Ciprofloxacin  TMP/SMX^2^  Amoxicillin-clavulanate | 5 days  3 days  3 days  5 days |  |
|  |  | ESBL | Nitrofurantoin | 5 days |  |
|  |  |  | Ciprofloxacin | 3 days |  |
|  |  |  | TMP/SMX | 3 days |  |
|  |  |  | Fosfomycin | 2 doses |  |
| Community-acquired – non-ICU Admission | Amoxicillin-clavulanate +  Azithromycin  OR Moxifloxacin | Pneumococcus  *Legionella*  Mycoplasma  *Haemophilus influenzae*  *Chlamydia pneumoniae*  *Moraxella catarrhalis* | Penicillin G  Azithromycin Doxycycline  Cefuroxime  Doxycycline  Cefuroxime | 5 – 7 days |  |
| Diverticulitis | Patients with uncomplicated diverticulitis (defined as CT confirmed left-sided disease without  abscess, free air or fistula ± fever, inflammatory markers, can be treated conservatively  without antibiotics. | | | | |
| Mild-Moderate infection   - If penicillin allergy (severe – IgE mediated) | Cefoxitin  Ciprofloxacin + Metronidazole | *Escherichia coli*  Enterobacterales  Streptococci  Enterococci  Anaerobes |  | 4 days unless adequate source control is not achieved |  |
| Mild Non-Purulent (cellulitis / erysipelas) | Cephalexin  OR  Flucloxacillin | Streptococci  Methicillin susceptible  *S. aureus*  MRSA | Cephalexin  Flucloxacillin  Doxycycline  OR  TMP / SMX  OR  Clindamycin | 1. days |  |

^1^Abridged version of our institutional guidelines with selected treatment recommendations based on the subset of patients encountered in the study.

^2^Trimethoprim / Sulfamethoxazole.
